# Kidney-Targeted Transplantation of Mesenchymal Stem Cells by Ultrasound-Targeted Microbubble Destruction Promotes Kidney Repair in Diabetic Nephropathy Rats

**DOI:** 10.1155/2013/526367

**Published:** 2013-05-27

**Authors:** Yi Zhang, Chuan Ye, Gong Wang, Yunhua Gao, Kaibin Tan, Zhongxiong Zhuo, Zheng Liu, Hongmei Xia, Dan Yang, Peijing Li

**Affiliations:** ^1^Department of Ultrasound, Xinqiao Hospital of the Third Military Medical University, 183 Xinqiaozheng Road, Chongqing 400037, China; ^2^Department of Ultrasound, The Forty-Fourth Military Hospital, 67 Huanghe Road, Guiyang 550009, China; ^3^Department of Orthopaedics, The Affiliated Hospital of Guiyang Medical College, 28 Guiyi Street, Guiyang 550004, China; ^4^Center for Tissue Engineering and Stem Cells, Guiyang Medical College, 9 Beijing Road, Guiyang 550004, China

## Abstract

We test the hypothesis that ultrasound-targeted microbubble destruction (UTMD) technique increases the renoprotective effect of kidney-targeted transplantation of bone-marrow-derived mesenchymal stem cells (BM-MSCs) in diabetic nephropathy (DN) rats. Diabetes was induced by streptozotocin injection (60 mg/Kg, intraperitoneally) in Sprague-Dawley rats. MSCs were administered alone or in combination with UTMD to DN rats at 4 weeks after diabetes onset. Random blood glucose concentrations were measured at 1, 2, 4, and 8 weeks, and plasma insulin levels, urinary albumin excretion rate (UAER) values, the structures of pancreas and kidney, the expressions of TGF-**β**1, synaptopodin, and IL-10 were assessed at 8 weeks after MSCs transplantation. MSCs transplantation decreased blood glucose concentrations and attenuated pancreatic islets/**β** cells damage. The permeability of renal interstitial capillaries and VCAM-1 expression increased after UTMD, which enhanced homing and retention of MSCs to kidneys. MSCs transplantation together with UTMD prevented renal damage and decreased UAER values by inhibiting TGF-**β**1 expression and upregulating synaptopodin and IL-10 expression. We conclude that MSCs transplantation reverts hyperglycemia; UTMD technique noninvasively increases the homing of MSCs to kidneys and promotes renal repair in DN rats. This noninvasive cell delivery method may be feasible and efficient as a novel approach for personal MSCs therapy to diabetic nephropathy.

## 1. Introduction

Both type 1 and type 2 diabetes mellitus (DM) involve destruction and dysfunction of pancreatic islets/*β* cells, and their main and long-term complication is diabetic nephropathy (DN) which has evolved as a leading cause of end-stage renal disease (ESRD). At the moment, transplantation of pancreatic islet and kidney is the most preferred cell replacement therapy to DN. However, the scarcity of transplantable donors and the need for lifelong immunosuppression limit the widespread use of the curative therapy.

Bone-marrow-derived mesenchymal stem cells (BM-MSCs), which possess multipotent differentiation characteristics, capacity for self-renewal, and immunomodulatory ability, are considered as a potential therapeutic agent for treatment of DN complications [1–4]. On the other hand, their utility for targeting tissue in living animals has proved to be limited. For instance, MSCs transplantation usually resulted in an insufficient number of engrafted MSCs in injury site. In view of the drawback, we have developed a technique that applies ultrasound-targeted microbubble destruction (UTMD) to promote homing of MSCs to impaired kidney.

Ultrasound contrast agent (microbubbles) is widely used to enhance the reflectivity of perfused tissues in clinical ultrasonography. Moreover, later researches focus on its potential therapeutic effect. The application of ultrasound to small vessels containing microbubbles can change blood vessel wall permeability, resulting in the extravasation of particles into the interstitial space [5]. In addition, UTMD has the potential to change the microenvironment [6], release the transported substances into target organ to repair damage tissue [7], and promote stem cells homing [8]. Currently, the majority of researchers consider that the interaction of ultrasound pulses with these gas bodies is a form of acoustic cavitation [9] and has successfully applied for blood vessels [10], skeletal muscle [11], heart [12], lung [13], liver [14], and tumors [15]. UTMD directed expression of an adenoviral reporter and was applied to selectively deliver plasmid vectors to the heart *in vivo* [16]. The transfection efficiency of cells *in vitro* was increased under the optimal UTMD conditions [17]. Lan et al. transferred a doxycycline-regulated Smad7 gene into the kidney using an ultrasound-microbubble-mediated system, specifically blocked TGF-*β* signaling and inhibited renal fibrosis in a rat unilateral ureteral obstruction (UUO) model [18]. Yu et al. suggested that the combined use of microbubble and high-intensity focused ultrasound (HIFU) improved the therapeutic efficiency of HIFU in rabbit kidney study [19]. Microbubble destruction by ultrasound gene transfection treatment (1.0 W/cm^2^) promoted renal recovery in acute kidney injury in rats [20]. So far, no studies have been reported whether this technique provides an equal contribution to diabetic kidney disease which acts as a complication of primary disease. Based on the above facts, we propose the hypothesis that UTMD is feasible for increasing the target transplantation of MSCs to kidney and promoting renal repair in diabetic nephropathy.

To test this hypothesis, MSCs (1 × 10^6^ cells) were administered alone or together with UTMD to DN rats at 4 weeks after diabetes onset. Normal nondiabetic rats were as those of control group. We then evaluated blood glucose concentrations, plasma insulin levels, UAER values, and the structure of kidney and pancreas, traced MSCs homing, accessed VCAM-1 levels after UTMD, and detected the levels of TGF-*β*1, IL-10, and synaptopodin after MSCs transplantation.

## 2. Materials and Methods

### 2.1. Animals

All Sprague-Dawley (SD) rats were provided by the Center for Experimental Animals of the Third Military Medical University. The experiments were approved by the Animal Care and Use Committee of Third Military Medical University. Rats were housed in wire cages with free access to a standard diet and tap water. The temperature and relative humidity of the animal facility were maintained under conventionally controlled conditions (22°C, 55% humidity) with a day-night rhythm.

### 2.2. Isolation and Culture of BM-MSCs

Three-week-old male SD rats were sacrificed by cervical dislocation. Bone marrow cells were obtained from femurs and tibias. After isolation and centrifugation, cells were cultured (37°C, 5% CO_2_) at a density of 1 × 10^6^ nucleated cells/cm^2^ with DMEM/F12 (Hyclone, Logan, UT, USA) medium containing 10% inactivated fetal bovine serum (Gibco, USA), 100 U/mL penicillin and 100 mg/mL streptomycin antibiotic solution (Gibco, USA). Medium was changed and nonadherent cells were removed after 72 hours. Adherent cells reaching 80% confluence were passaged with 0.25% trypsin-EDTA solution (Gibco, USA) and then subcultured in DMEM/F12 medium with 10% inactivated fetal bovine serum, 100 U/mL penicillin, and 100 mg/mL streptomycin antibiotic solution (complete medium).

### 2.3. Adipogenic, Osteogenic, and Chondrogenic Differentiation of MSCs

Adipogenic differentiation was induced by culturing MSCs with 10 nM dexamethasone (Sigma) and 5 *μ*g/mL insulin (Sigma) for 7 days with medium change every 3 days. The intracellular lipid droplets were easily observed by phase-contrast microscopy and assessed using Oil Red O (Sigma) staining.

Osteogenic differentiation was induced by culturing MSCs with 10 nM dexamethasone (Sigma), 50 *μ*g/mL ascorbic acid (Sigma), and 10 mM *β*-glycerol phosphate (Sigma) for 2 weeks with medium change every 3 days. The calcium deposits were stained with alizarin red S.

Chondrogenic differentiation was induced by culturing MSCs with 10 ng/mL rh-TGF*β*1 (Sigma), 50 *μ*g/mL ascorbic acid (Sigma), 6.25 mg/mL insulin (Sigma), 10^−7^ M dexamethasone (Sigma), and 12 *μ*M L-glutamine (Sigma) for 7 days with medium change every 3 days. The differentiated cells were assessed using Safranin O (Sigma) staining.

### 2.4. Flow Cytometry Analysis of MSCs

Approximately 1 × 10^6^ MSCs were prepared and washed 2 times by centrifugation at 900 ×g for 5 min. MSCs were then resuspended in 500 *μ*L of PBS and incubated with phycoerythrin- or fluorescein isothiocyanate-conjugated antibodies against rat CD34, CD44, CD45, and CD90 for 30 min at 4°C. All assays were conducted according to the manufacturer's instructions. Phycoerythrin-conjugated mouse anti-rat IgG1 was used as an isotype control. Cells were collected and washed with PBS by centrifugation and fluorescence analysis was carried out with a flow cytometer (Beckman, USA).

### 2.5. MSCs Labeling

To track the transplanted MSCs in kidney, enhanced green fluorescent protein (eGFP, Cyagen Biosciences) was used as a cell tracker. MSCs were transfected with lentiviral vectors carrying eGFP. Briefly, MSCs after the third passage were seeded at 1 × 10^5^ cells in 6-well plates 24 hours before transfection. The next day, 1 mL complete medium per well containing 5 *μ*g/mL polybrene (Sigma) and viral were added to the cells at a MOI (multiplicity of infection) of 10, and then cells were incubated at 37°C in 5% CO_2_ for 8 hours. After incubation, cells were washed with PBS and incubated in fresh complete medium for further experimentation. 72 hours after transfection, the infected cells were observed by fluorescent microscope. 

### 2.6. Diabetic Nephropathy Induction

Adult male SD rats aged 7-8 weeks (160–180 g body weight) were used. All rats were lightly anesthetized by intraperitoneal injection of 2% pentobarbital sodium at a dose of 35 mg/kg and received a single intraperitoneal injection of 60 mg/kg streptozotocin (STZ, Sigma) immediately after dissolving in 0.1 M citrate buffer at pH 4.5. Three days after STZ injection, diabetes was confirmed by random blood glucose concentrations of greater than 16.7 mmol/L for 3 consecutive days. 4 weeks after diabetes onset, diabetic rats presented mild microalbuminuria (an early sign DN) and were ready for further experiment.

### 2.7. Experimental Preparation

The microbubbles used in this study were self-made, which was a type of perfluoropropane-gas-filled microbubbles with lipid shell and was awarded by the National Invention Patent of China in 2005. Its concentration and mean diameter were approximately 4–9 × 10^9^/mL and 2.13 *μ*m, respectively. A recent study confirmed that it did not produce changes in right ventricular blood pressure, had no significant influence on rat kidney, and better enhanced the ultrasound imaging, showing a fine properties and prospects for application [21]. The administration dosage of microbubbles for each injection was 0.05 mL/kg, and microbubbles were injected into tail vein through 26-gauge needles connected with a 1 mL syringe via a 15 cm length catheter, followed by 0.5 mL saline to wash the tube. MSCs (1 × 10^6^) were resuspended in 2 mL of PBS and administrated to anesthetized rats through tail vein. A color diagnostic ultrasound system (S2000, Siemens, Germany) was applied, a high-frequency probe of 9L4 was used, and the parameters were set as follows: center frequency of 7.00 MHz; mechanical index of 0.9; continuous irradiation; depth of 3 cm; and single focal point of 1 cm. Rats were fixed in left lateral position after satisfactory anesthesia by intraperitoneal injection of 2% pentobarbital sodium at a dose of 40 mg/kg and only the right kidney was irradiated. Right flank was shaved with a shaver, and the remaining hair was removed using a depilatory cream. Ultrasound probe was put to the right kidney and irradiated for 5 min.

### 2.8. Real-Time Polymerase Chain Reaction (Real-Time PCR) and Transmission Electron Microscopy (TEM)

DN rats (*n* = 32) were randomly divided into four groups and received MSCs transplantation: (1) DN rats received 2 mL of PBS (phosphate-buffered saline) infusion (PBS group); (2) DN rats received ultrasonic irradiation together with microbubbles infusion (UTMD group); (3) DN rats received MSCs infusion (MSCs group); and (4) DN rats received ultrasound + microbubbles combined with MSCs infusion (UTMD + MSCs group). Three days after MSCs transplantation, rats were killed by anesthetic overdose and kidneys were rapidly dissected out. Real-time PCR analysis was performed to investigate VCAM-1 mRNA expression, and renal capillary permeability was observed by transmission electron microscopy.

#### 2.8.1. Real-Time PCR

The whole tissue preparation procedure was carried out at 4°C. Renal cortices were weighted, minced, and homogenized in a glass homogenizer. DNA was extracted with chloroform and precipitated with ethanol, and total DNA was assayed by UV absorbance. The oligonucleotide primers were as follows: VCAM-1, 5′-CGTTGACATCCGTAAAGACCTC-3′ (sense), and 5′-TAGGAGCCAGGGCAGTAATCT-3′ (antisense). *β*-actin, 5′-GCGAGGTCGTTTAGAGTAGTAGC-3′ (sense), and 5′-CCTGAAAGTCAACCCAGTGA-3′ (antisense). Real-time PCR assay was performed with target DNA, VCAM-1 primers, and a fluorescent probe by using a real-time PCR instrument (Applied Biosystems).

#### 2.8.2. TEM

Targeted homing ability of MSCs to kidneys was associated with renal capillary permeability which was assessed by TEM. Renal cortices were cut into 1 mm pieces and fixed in 2% glutaraldehyde for 1 hour. After being washed with PBS for 3 times, the samples were postfixed in 1% osmium tetroxide in cacodylate buffer (pH 7.2) for 1 hour. Subsequently, the samples were dehydrated in ethanol and embedded in epoxy resin (Agar 100). Ultrathin sections (50 nm) were cut, double stained with uranyl acetate and Reynolds lead citrate, and examined with a transmission electron microscope (Philips TECNAI 10, USA).

### 2.9. MSCs Tracking

DN rats (*n* = 10) were equally divided into MSCs group and UTMD + MSCs group. Three days after cells transplantation, rats were killed by anesthetic overdose. Kidneys and pancreases were separated and immediately snap-frozen in liquid nitrogen for frozen sections (5 *μ*m in thickness). Nuclear counterstaining was performed with 4′,6-diamidino-2-phenylindole (DAPI, Beyotime, China). The survival of implanted MSCs was observed and evaluated in frozen sections by counting five randomly chosen fields under laser scanning confocal microscope (LSCM) from each rat in the two groups. 

### 2.10. Allogenic MSCs Implantation

**Figure 1:**
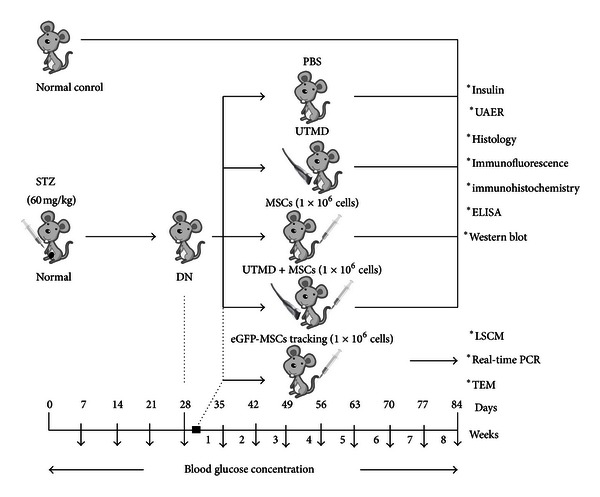
The experimental protocol. Four weeks after diabetes onset induced by STZ (60 mg/Kg), rats presented mild microalbuminuria (DN rats) and divided into PBS group (PBS infusion), UTMD group (ultrasound + microbubble), MSCs group (1 × 10^6^ eGFP labeled MSCs for infusion), and UTMD + MSCs group (MSCs infusion combined with ultrasound and microbubble), and a group of normal nondiabetic rats (no treatment) was set as a normal control group. Random blood glucose concentrations were measured at 1, 2, 4, and 8 weeks, and determination of plasma insulin, UAER, histology, immunofluorescence, immunohistochemistry, ELISA, and Western blot were performed 8 weeks after MSCs treatment. To track the intrarenal localization of implanted MSCs and explore the underlying mechanism, DN rats which received ultrasonic irradiation with or without eGFP-labeled MSCs infusion were killed 3 days after treatment, and laser scanning confocal microscope (LSCM), Real-Time PCR, and transmission electron microscopy were performed.

Figure 1 showed the experimental protocol. DN rats (*n* = 40) were randomly divided into four groups (*n* = 10/group) and a group of normal nondiabetic rats (*n* = 10) was set as a normal control group. Rats of five groups received following treatment: (1) normal nondiabetic rats without treatment (normal group); (2) DN rats received 2 mL of PBS infusion (PBS group); (3) DN rats received ultrasonic irradiation together with microbubbles infusion (UTMD group); (4) DN rats received MSCs infusion (MSCs group); (5) DN rats received ultrasound + microbubbles combined with MSCs infusion (UTMD + MSCs group). All rats of five groups were housed in wire cages for 8 weeks after treatment. Random blood glucose concentrations were measured at 1, 2, 4, and 8 weeks, plasma insulin levels, UAER values, the structures of pancreas and kidney, immunohistochemistry for TGF-*β*1, ELISA for IL-10, and Western blot for synaptopodin were assessed at 8 weeks.

### 2.11. Blood Glucose Concentrations Determination

Blood samples were collected from the tail vein from nonfasted rats and blood glucose concentrations were determined by a glucometer (Accu-Chek Aviva; Roche Applied Science).

### 2.12. Plasma Insulin Levels Determination

Plasma insulin levels were assayed by a radioimmunoassay (RIA) kit (Atom High-Tech Co., Ltd., Beijing, China), and the procedure was carried out in accordance with the manufacturer's instructions.

### 2.13. UAER Values Determination

UAER values were determined using the albumin-to-creatinine ratio (ACR) in 24-hour urine collections. The concentrations of urine albumin and urine creatinine were determined in Xinqiao Hospital clinical laboratory using an automatic biochemical analyzer (Hitachi, Japan).

### 2.14. Pancreas Double-Label Immunohistofluorescence

Deparaffinized pancreatic sections were incubated for 2 hours with monoclonal mouse anti-rat insulin and polyclonal rabbit anti-rat glucagon from Boster (China). After washing, the sections were incubated for 1 hour with TRITC-conjugated anti-mouse IgG and FITC-conjugated anti-rabbit IgG from Boster (China). Cross-reactivity of secondary antibodies was performed by control experiment of omitting primary antibodies. Slices were examined under laser scanning confocal microscope, and nuclear counterstaining was visualized with 4′,6-diamidino-2-phenylindole (DAPI, Beyotime, China).

### 2.15. Kidney and Pancreas Histology

At the end of the experiment, rats were sacrificed by anesthetic overdose. Kidneys and pancreases were rapidly removed, fixed in 10% formaldehyde, embedded in paraffin, and sectioned at 5 *μ*m thickness. Renal sections were stained with periodic acid-Schiff (PAS) staining and pancreatic sections were stained with hematoxylin-eosin (H&E) staining. All sections were evaluated under a light microscope by a pathologist who was blinded to treatment.

### 2.16. Immunohistochemistry

Expression of TGF-*β*1 (transforming growth factor-*β*1) in renal tissue was assessed by immunohistochemistry. Renal sections were deparaffinized, rehydrated, and submitted to microwave antigen retrieval in citrate buffer, pH 6.0, at 95°C for 10 min. The endogenous peroxidase activity was blocked with 3% H_2_O_2_ for 10 min, and sections were blocked with normal goat serum for 10 min. Then, the sections were incubated with rabbit polyclonal TGF-*β*1 (Boster, China) antibody or a negative control reagent at 4°C, followed by biotinylated anti-rabbit IgG secondary antibody for 30 min at 37°C. Antigen-antibody reactions were visualized with diaminobenzidine (DAB) which resulted in a brown-colored precipitate at the antigen site, and hematoxylin counterstaining was performed. Five random fields of each section were photographed at a magnification of 400x and semiquantitative evaluations were assessed using Image-Pro Plus 6.0 software.

### 2.17. ELISA

Renal cortices were placed in cell lysis buffer and protease inhibitors on ice. Samples were homogenized and homogenates were centrifuged at 15,000 rpm for 15 min at 4°C. The amount of IL-10 in supernatants was measured using enzyme-linked immunosorbent assay (ELISA, R&D Systems, Minneapolis, MN, USA) according to the manufacturer's instructions.

### 2.18. Western Blot

Renal cortices were homogenized in lysis buffer on ice for 30 minutes. Protein samples were separated by sodium dodecyl sulfate-polyacrylamide gel electrophoresis and then transferred to polyvinylidene fluoride (PVDF) membranes. Membranes were blocked using 0.1% Tween-20 in Tris-buffered saline (TTBS) containing 5% bovine serum albumin (BSA) at room temperature for 2 hours. Then, membranes were washed three times with TTBS and incubated for 2 hours at room temperature with rabbit polyclonal antisynaptopodin (Abcam, 1 : 500 in TTBS) and *β*-Actin (stained by polyclonal anti-*β*-Actin 1 : 500 dilution, Abcam). The signal was detected by chemiluminescence method (PIERCE).

### 2.19. Statistical Analysis

Data are summarized as mean ± standard deviation for each group. Student's *t*-test was used to determine the significant difference between two groups. One-way ANOVA and post hoc comparisons (Bonferroni's test) were used to determine the significant difference among multiple groups. A *P* value of less than 0.05 was considered to be statistically significant.

## 3. Results

### 3.1. Characteristics, Identification, and Labeling of MSCs

**Figure 2:**
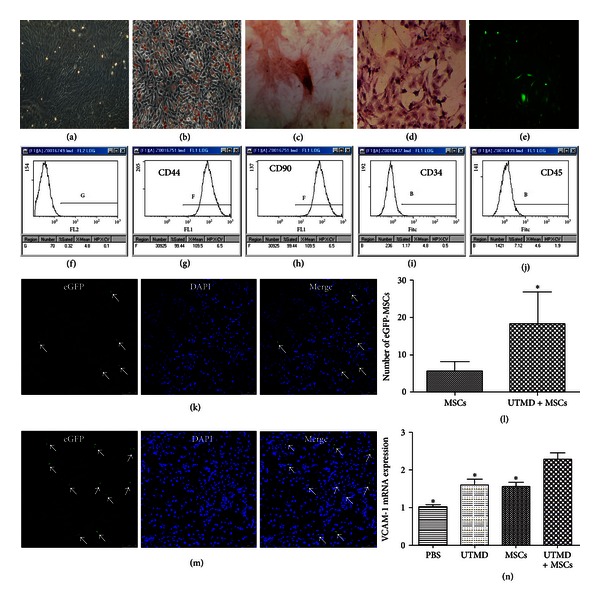
BM-MSCs culture, characteristics, and tracking. (a) After 10–14 days of primary culture, MSCs were nearly 80%–90% confluent (×100). The intracellular lipid droplets stained with oil red O staining (b), the calcium depositions stained with alizarin red S staining (c), the chondrogenic cells stained with Safranin O staining (d) were observed after 1 week and 2 week of treatment, respectively. (e) MSCs transfected by lentiviral vectors carrying eGFP showed bright green fluorescence. The phenotype of rat MSCs was shown to be positive for CD44 (99.44%, (g)), CD90 (99.37%, (h)), and negative for CD34 (1.17%, (i)), CD45 (7.12%, (j)). (f) represents the isotype control. There were much more eGFP-labeled MSCs localized in the kidney in UTMD + MSCs group (m) than those in MSCs group (k). (l) Comparison of the eGFP-labeled MSCs between MSCs group and UTMD + MSCs group, *P < 0.01 versus groups, independent *t*-test. (n) VCAM-1 mRNA expression was increased by UTMD and/or MSCs infusion and it had a significant increase after MSCs infusion together with UTMD, *P < 0.01 versus groups.

Most of cultured MSCs were spindle shaped, attached to the culture dish tightly, proliferated in culture medium, and became more uniform after several passages (Figure 2(a)). Adipogenic, osteogenic, and chondrogenic differentiation were successfully induced in MSCs after 1 week and 2 weeks of treatment, respectively. The intracellular lipid droplets, calcium depositions and chondrogenic cells were observed by phase-contrast microscope (Figures 2(b)–2(d)). Flow cytometry analysis showed that these expanded MSCs were positive for CD44 (99.44%, Figure 2(g)), CD90 (99.37%, Figure 2(h)), and negative for the leukocyte common antigens CD34 (1.17%, Figure 2(i)), CD45 (7.12%, Figure 2(j)).

MSCs transfected by lentiviral vectors carrying eGFP showed bright green fluorescence 72 hours after transfection (Figure 2(e)) under fluorescence microscopy. It was worth noting that eGFP-MSCs mostly localized in outer region of interstitial capillary, the peritubular area (Figures 2(k) and 2(m)). A small number of eGFP-MSCs located in glomeruli and approximately 10% glomeruli presented about 1 to 3 eGFP-MSCs. Quantification analysis of eGFP-MSCs in renal tissue showed that there was a significant difference between MSCs group and UTMD + MSCs group. The number of eGFP-MSCs in UTMD + MSCs group (18.3 ± 2.9, Figure 2(m)) was much more than that of MSCs group (5.7 ± 0.8, Figure 2(k)). Figure 2(l) represents the histogram of the quantification analysis. No MSCs were found in pancreas.

### 3.2. VCAM-1 mRNA Expression

VCAM-1 mRNA expressions in each group are shown in Figure 2(n). The result revealed that VCAM-1 mRNA expression was increased both in UTMD group and MSCs group and it was markedly increased in UTMD + MSCs group compared with PBS group (*P* < 0.01), UTMD group (*P* < 0.01), and MSCs group (*P* < 0.01).

### 3.3. Increase of Interstitial Capillary Permeability

**Figure 3:**
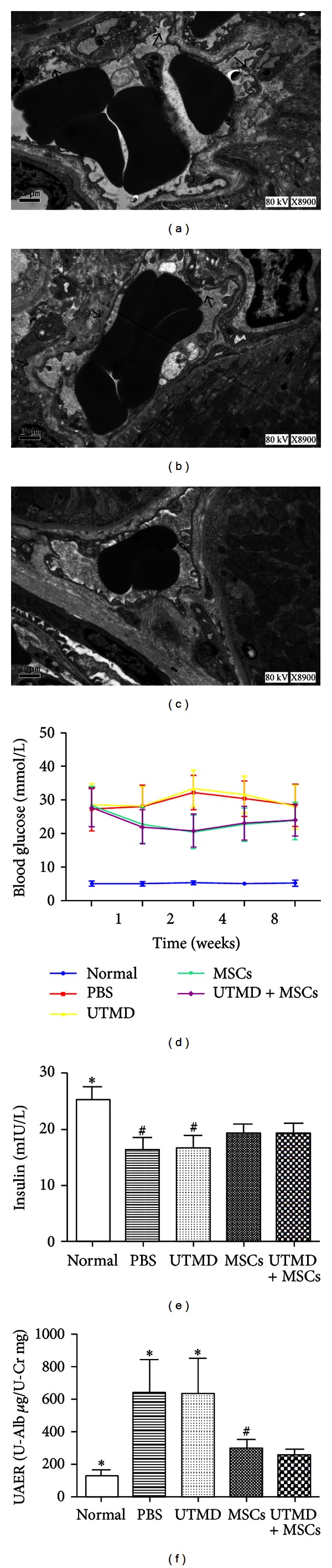
The endothelial cells of renal interstitial capillary were mildly injured under TEM and a part of interstitial capillary walls became thinner, discontinuous, and roughened in UTMD group ((a), arrow) and UTMD + MSCs group ((b), arrow), while the endothelial cells and interstitial capillary walls were kept intact in PBS group and MSCs group (c). (d) A trend graph for the change of blood glucose concentrations at 1, 2, 4, and 8 weeks after MSCs transplantation alone or together with UTMD. (e) 8 weeks after cell therapy, plasma insulin levels increased in MSCs group and UTMD + MSCs group, compared with PBS group and UTMD group, respectively. *P < 0.01 versus groups and ^#^ *P* < 0.05 versus groups. (f) MSCs transplantation with or without UTMD significantly decreased the increased UAER values of PBS group and UTMD group, and there was a statistical difference between MSCs group and UTMD + MSCs group. *P < 0.01 versus groups and ^#^ *P* < 0.05 versus groups.

Transmission electron microscope was used to observe the ultrastructural change of renal capillary, and we found that a part of interstitial capillary walls became thinner, discontinuous, and roughened in UTMD group and UTMD + MSCs group (Figures 3(a) and 3(b)) and they kept intact in PBS group and MSCs group (Figure 3(c)), suggesting that a mild injury of endothelial cells was responsible for the changes and interstitial capillary permeability was increased by UTMD. 

### 3.4. The Changes of Blood Glucose Concentrations, Plasma Insulin Levels, and UAER Values

In order to observe the improvement of pancreatic damage, blood glucose concentrations and plasma insulin levels were assessed. Four weeks after STZ injection, blood glucose concentrations of DN rats increased from normal level (5.2 ± 0.7 mmol/L, normal group) to severe hyperglycemia (27.3 ± 6.5 mmol/L, PBS group). Within 1 week after cell therapy, blood glucose concentrations significantly decreased in MSCs group (22.5 ± 5.3 mmol/L) and UTMD + MSCs group (22.1 ± 5.0 mmol/L), reached the lowest levels at 2 weeks, and lasted at least during the observation period (Figure 3(d)). In contrast, untreated PBS group (28.4 ± 6.2 mmol/L) and UTMD group (27.9 ± 6.5 mmol/L) remained a high level of blood glucose until the end of the experiment. No obvious difference was found in blood glucose concentration between MSCs group (23.8 ± 5.5 mmol/L) and UTMD + MSCs group (24.0 ± 4.6 mmol/L), *P* = 0.395, which was the same as that between PBS group (28.4 ± 6.2 mmol/L) and UTMD group (27.9 ± 6.5 mmol/L), *P* = 0.816.

The increased blood glucose concentrations were associated with the deceased plasma insulin levels. Compared with normal group (25.27 ± 2.34 mIU/L), plasma insulin levels decreased in PBS group (16.42 ± 2.17 mIU/L) and UTMD group (16.67 ± 2.33 mIU/L) and improved in MSCs group (19.31 ± 1.68 mIU/L) and UTMD + MSCs group (19.53 ± 1.59 mIU/L) (Figure 3(e)). There was no significant difference in plasma insulin levels between MSCs group and UTMD + MSCs group (*P* = 0.574), which was the same as that between PBS group and UTMD group (*P* = 0.564).

Rats displayed mild microalbuminuria 4 weeks after STZ injection and UAER values of DN rats increased from 57.79 ± 13.42 *μ*g/mg (normal group) to 85.47 ± 19.43 *μ*g/mg (PBS group). At the end of the experiment, UAER values maintained a high level in PBS group (643.25 ± 204.58 *μ*g/mg) and UTMD group (637.29 ± 212.24 *μ*g/mg), and both of them were more than fourfold higher than that of age-matched normal control rats (128.57 ± 36.93 *μ*g/mg) (Figure 3(f)). As a comparison, UAER values reduced in MSCs group (302.41 ± 49.21 *μ*g/mg) and UTMD + MSCs group (252.83 ± 39.58 *μ*g/mg). Furthermore, UAER values were significantly milder in UTMD + MSCs group (252.83 ± 39.58 *μ*g/mg) than that in MSCs groups (302.41 ± 49.21 *μ*g/mg). Although UAER values in MSCs group and UTMD + MSCs group never reached the normal level, UTMD + MSCs group showed an obvious fall in the level than MSCs group.

### 3.5. Pathological Changes of Pancreas and Kidney

**Figure 4:**
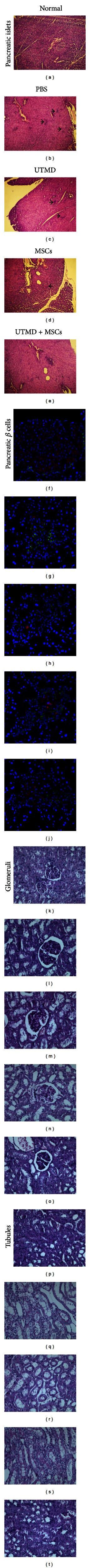
(a–e) Pancreatic histological sections were stained with H&E staining and observed under light microscope. Pancreatic islets structures were indicated by black arrows (b–d). (f–j) The amounts of insulin- and glucagon-producing cells (red and green, resp.) were observed by pancreatic double-label immunohistofluorescence. (k–t) Renal histological sections were stained with PAS staining and observed under light microscope. Normal rats glomeruli (k) and tubules (p). Glomerular sclerosis and glomerular mesangial expansion (l and m) and tubular dilatation (q and r) were observed in PBS group and UTMD group. MSCs-treated rats glomeruli (n) and tubules (s). Even nearly normal structure of glomeruli (o) and tubules (t) were observed in UTMD + MSCs group.

At the end of the experiment, PBS group and UTMD group showed a massive destruction of pancreatic islets/*β* cells observed by pancreatic pathology and double-label immunohistofluorescence. Compared to normal pancreatic islets/*β* cells (Figures 4(a) and 4(f)), morphological irregularity, volume reduction, and less insulin- and more glucagon-positive cells were observed in PBS group and UTMD group (Figures 4(b), 4(c), 4(g), and 4(h)). As expected, MSCs group and UTMD + MSCs group exhibited a good recovery in amount, architecture and volume of pancreatic islets/*β* cells (Figures 4(d), 4(e), 4(i), and 4(j)).

Renal histological analysis showed that there have been similar changes both in PBS group and UTMD group in glomeruli and tubules which displayed glomerular sclerosis, mesangial expansion, and tubular dilatation observed by light microscope (Figures 4(l), 4(m), 4(q), and 4(r)). However, the extent of such changes of glomeruli and tubules was extenuated in MSCs group and UTMD + MSCs group (Figures 4(n), 4(o), 4(s), and 4(t)). Only a small part of glomerular hyalinosis and tubular dilatation were observed in MSCs group. Further improvements in structure or even nearly normal structure of glomeruli and tubules were observed in UTMD + MSCs group.

### 3.6. Immunohistochemistry

**Figure 5:**
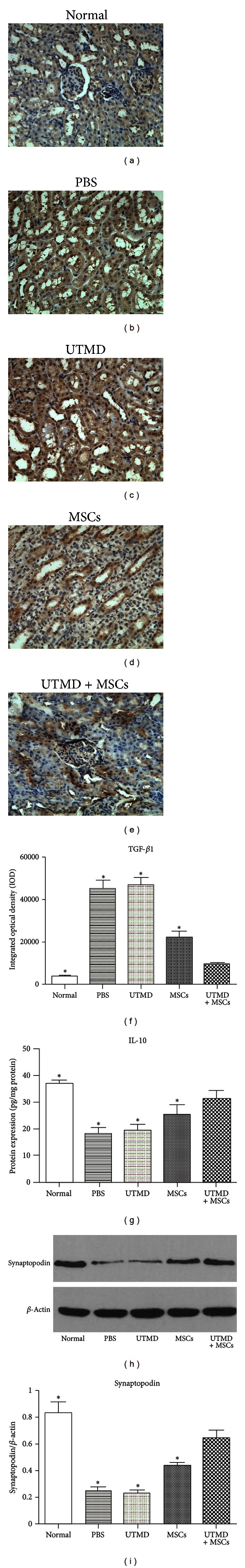
(a–e) Immunohistochemical staining of TGF-*β*1 in kidney of normal group (a), PBS group (b), UTMD group (c), MSCs group (d), and UTMD + MSCs group (e). The brown area was the positive expression of TGF-*β*1 and TGF-*β*1 expression significantly decreased after MSCs transplantation combined with UTMD technique. *P < 0.01 versus groups. (f) represents the histogram of integrated optical density (IOD) of TGF-*β*1 expression analyzed using Image-Pro plus 6.0 software. (g) IL-10 levels in renal cortices were determined by ELISA and they were obviously higher in UTMD + MSCs group than those in PBS group, UTMD group, and MSCs group. *P < 0.01 versus groups. (h and i) represents Western blot (h) and summary of densitometric analyses (i) for synaptopodin expression, which showed that synaptopodin expression increased after MSCs infusion and there was a greater improvement in UTMD + MSCs group. *P < 0.01 versus groups.

TGF-*β*1 was slightly expressed in glomeruli and tubules in normal group (Figure 5(a)) and strongly expressed in tubules in PBS group (Figure 5(b)) and UTMD group (Figure 5(c)). TGF-*β*1 expression decreased in MSCs group (Figure 5(d)) and UTMD + MSCs group (Figure 5(e)) after MSCs administration, and a further decrease was observed in UTMD + MSCs group.  Figure 5(f) represents the quantification analysis of integrated optical density (IOD) of pictures of five groups.

### 3.7. ELISA

As shown in Figure 5(g), renal cortical IL-10 levels significantly decreased in PBS group (18.24 ± 2.17 pg/mg protein) and UTMD group (19.32 ± 2.25 pg/mg protein), compared with nNormal group (36.92 ± 1.27 pg/mg protein). After MSCs treatment, IL-10 levels notable increased in MSCs group (25.43 ± 3.46 pg/mg protein) and UTMD + MSCs group (31.15 ± 3.19 pg/mg protein), compared with PBS group (18.24 ± 2.17 pg/mg protein). IL-10 levels in UTMD + MSCs group (31.15 ± 3.19 pg/mg protein) were higher than those in MSCs group (25.43 ± 3.46 pg/mg protein).

### 3.8. Western Blot

Consistent with the result of ELISA, synaptopodin protein expression in renal cortices decreased in PBS group and UTMD group, compared with normal group. After MSCs implantation, synaptopodin protein expression increased both in MSCs group and UTMD + MSCs group, and a further improvement in UTMD + MSCs group was detected.

## 4. Discussion

Ultrasound contrast agent (microbubbles) has developed to produce intense echoes and enhance blood-to-tissue contrast in clinical ultrasonography. Moreover, microbubbles destruction under ultrasonic irradiation has been proposed as a new, appealing technique for site-specific drug and gene delivery *in vitro* and *in vivo *and has been introduced in a variety of application areas.

Mesenchymal stem cells (MSCs) are the stromal component of bone marrow (BM) and easily obtained from BM, have the potential to differentiate into several cell types, and show immunomodulatory properties. MSCs are capable of differentiating into functional insulin-producing cells *in vitro*, which can reverse hyperglycemia in diabetes rats [22]. MSCs also have the potential to differentiate into renal cells *in vivo*, which can repair the destroyed kidney [23]. Therefore, MSCs are regarded as an attractive strategy to ameliorate hyperglycemia and improve renal function [24, 25] through intracardiac injection, intra-arterial or intravenous injection, and intrarenal injection.

The use of MSCs for cell therapies relies on the capacity of these cells to home and engraft in the long term into the appropriate target tissue [26]. In this experiment, we showed here that the number of eGFP-labeled MSCs in the kidney of UTMD + MSCs group was significantly larger than those of MSCs group, which demonstrated that UTMD has the capability of increasing tropism of MSCs to damage kidney tissue. To prove this point of view, transmission electronic microscope was performed to observe the ultrastructure of renal capillary after ultrasonic irradiation and we found that parts of interstitial capillary walls became thinner, discontinuous, and roughened after UTMD. It directly proved that interstitial capillary permeability was increased by UTMD, which facilitated the homing and gathering of MSCs to target tissue. VCAM-1 mRNA expression was performed after UTMD to clarify its underlying mechanism. The result suggests that VCAM-1 expression increases after MSCs implantation or UTMD, while a higher expression of VCAM-1 was shown in UTMD + MSCs group. It means that paracrine effect of the transplanted MSCs or ultrasound-targeted microbubble destruction leads to the upregulation of adhesion molecule, respectively. The combined effect of UTMD and MSCs induces a higher expression of VCAM-1 on the targeted vascular endothelium in UTMD + MSCs group, which causes the enhanced attachment of transfused MSCs onto the targeted endothelial layer [8]. Therefore, the targeted delivery and the enhanced homing of implanted MSCs to kidney might be induced by the increased interstitial capillary permeability under UTMD and the higher expression of VCAM-1 caused by acoustic cavitation of ultrasound and paracrine effect of MSCs.

No significant difference was found between MSCs group and UTMD + MSCs group in blood glucose concentration, the regeneration of insulin cells, and the structural recovery of pancreatic islets. A possible explanation was that only kidney, not pancreas, received ultrasonic irradiation before MSCs transplantation. There was no difference in the state of pancreatic vessels before and after MSCs transplantation. Therefore, there might be an equivalent amount of MSCs engrafted into pancreatic tissue and might have an equal therapeutic effect in MSCs group and UTMD + MSCs group. This phenomenon confirmed that UTMD played the role of specific target transplantation at the same time.

There is now strong evidence that the onset and progression of diabetic complications is significantly delayed by improving glycaemic control [27]. MSCs administration results in the reduction of blood glucose levels and prevents renal damage in diabetic mice [28]. Although no MSCs were found in pancreas, our experiment shows that MSCs transplantation reduces blood glucose concentrations, enhances insulin levels, and improves the morphology, structure, and quantity of pancreatic islets/*β* cells. MSCs acting through paracrine action might be a reasonable interpretation. In addition, as the kidneys receive approximately 25% of the cardiac output, therefore they have a richer blood supply than pancreas. This means that implanted MSCs through tail vein have more chance to engraft into renal interstitium, and thus it might be relatively easier to track eGFP-labeled MSCs in kidney than in pancreas. There was a significant difference in UAER value, the pathological change of kidneys, and renal cortical cytokine and protein expression after MSCs treatment between MSCs group and UTMD + MSCs group, and there was almost no apparent difference in these observed items between PBS group and UTMD group. This was possible for three reasons: (1) recent studies confirmed that MSCs crossed the endothelial cell layer and recruited to damage tissues analogous to those of inflammatory cells. In our study, interstitial capillary permeability in renal tubulointerstitial area and renal cortical VCAM-1 levels were increased by UTMD, which made MSCs more easily to attach and cross the thinner and discontinuous capillary walls and gather to damage kidney; (2) correspondingly, UTMD used alone may have negligible impact on the tissue repair for no MSCs supply in time during the change of permeability and the levels of adhesion molecule; (3) the reduction of blood glucose concentration in MSCs-treated rats depends on MSCs therapy. Renoprotection in MSCs-treated rats is correlated not only with MSCs therapy, but also with an improvement of endocrine pancreatic function. That is to say, the lower blood glucose concentrations play a facilitative effect for protecting the kidney. To sum up, there are more MSCs homing, retention, and participation for renal therapy after UTMD than MSCs transplantation alone. MSCs transplantation in combination with UTMD was not only a noninvasive method for cell-targeted delivery, but also an efficient approach for cell therapy.

TGF-*β* is a prototypical hypertrophic and fibrogenic cytokine [29]. It stimulates renal cell hypertrophy and extracellular matrix accumulation which are two hallmarks of diabetic nephropathy. It causes glomerular basement membrane (GBM) thickening and may promote podocyte apoptosis or detachment [30]. Cellular hypertrophy and matrix production are stimulated by high glucose concentrations in tissue culture studies. High glucose, in turn, appears to act through the TGF-*β* system because high glucose increases TGF-*β* expression, and the hypertrophic and matrix-stimulatory effects of high glucose are prevented by anti-TGF-*β* therapy [31]. The role of MSCs in antifibrotic therapy is still a matter of controversy. Ezquer et al. showed that MSCs administration prevented the onset of nonalcoholic steatohepatitis in obese mice [32]. However, Carvalho et al. indicated that MSCs were unable to reduce fibrosis or improve liver function in a rat model of severe chronic liver injury [33]. TGF-*β*1 is considered the most fibrogenic isoform of TGF-*β*. In our finding, MSCs transplantation prevents the development of renal hypertrophy, mesangial matrix expansion, and tubular dilatation, and decreases TGF-*β*1 expression, which means that glomerulosclerosis and renal interstitial fibrosis are prevented effectively. It might be correlated both with the decrease of blood glucose and with the inhibition of TGF-*β*1 expression by MSCs.

IL-10, the main anti-inflammatory cytokine and immunosuppressive cytokine, is produced by several types of immune cells such as T regulatory and Th2 lymphocytes, activated macrophages, B regulatory lymphocytes as well as other cell types [34]. It plays a key role in the regulation of immune responses, has a potent deactivator of monocyte/macrophage proinflammatory cytokine synthesis, and inhibits leukocyte infiltration, inflammation, and tissue damage associated with immunological response [35]. Diabetic nephropathy is an inflammatory disease and accumulating evidence now indicates that immunologic and inflammatory mechanisms play a significant role in its development and progression [36, 37]. Inflammatory factors promote macrophage accumulation and activation and accelerate the progression of diabetic renal injury [38]. Therefore, IL-10, as an immunoregulatory cytokine, may be correlated with diabetic nephropathy and its anti-inflammatory properties have been similarly demonstrated in animal models [39]. In our model, DN rats were manifest by albuminuria, glomerular hypertrophy, tubular dilatation, and an apparent reduction of IL-10 amount, while most manifestations were ameliorated after MSCs transplantation. These data suggest that MSCs transplantation attenuates inflammatory response of kidney in diabetic nephropathy, leads to modulation of the inflammation through upregulation of IL-10 cytokine, and delays the progression of diabetic nephropathy. It also shows that IL-10 is involved in the pathogenesis of diabetic nephropathy and the attenuation of inflammation could mitigate renal interstitial injury. This beneficial effect is thought to be due to the anti-inflammatory factors, such as IL-10, produced by MSCs that are secreted in a paracrine fashion [39].

Although the quantity of implanted MSCs in glomeruli were relatively less than that in renal interstitium, glomerular repair was obvious as well. Podocyte lines the outer aspect of glomerular basement membrane (GBM), therefore forming the final barrier to protein loss [40]. Podocyte injury has been suggested to be associated with the pathogenesis of albuminuria in diabetic nephropathy and predicts the progressive course of diabetic nephropathy. Synaptopodin is a proline-rich, actin-associated protein which may play an important role in modulating podocyte foot processes (FP), and highly expressed in telencephalic dendrites and renal podocytes [41, 42]. From this experiment, podocytes repair was assessed by detecting synaptopodin protein expression and UAER values. The results show that UAER values significantly increase and synaptopodin protein expression significantly decreases 8 weeks after diabetes onset, indicating a serious injury in podocyte. MSCs transplantation ameliorates podocyte injury for decreasing UAER values and increasing synaptopodin expression. Furthermore, kidney-targeted transplantation of MSCs mediated by UTMD showed a better improvement in UAER values and upregulated the more synaptopodin expression, suggesting an apparent podocyte repair.

In this paper, we provide an intuitive proof (transmission electron microscope) to prove that ultrasonic cavitation increases interstitial capillary permeability, which facilitates the homing of MSCs to injured kidney. UTMD technique combined with MSCs transplantation induces a higher expression of the adherent molecule (VCAM-1), which promotes the attachment and gathering of MSCs to interstitial capillary endothelium of damage kidney. Target transplanted MSCs secrete anti-inflammatory cytokine (IL-10) by a paracrine action, which leads to the increase of IL-10 levels and the reduction of inflammatory response. Infused MSCs repair the podocyte and renal interstitial injury by upregulating synaptopodin expression and downregulating TGF-*β*1 expression, resulting in that the manifestations such as glomerular sclerosis, mesangial expansion, and tubular dilatation were obviously improved. 

In conclusion, MSCs transplantation reverts hyperglycimia and kidney-targeted transplantation of MSCs mediated by UTMD increases MSCs homing to damage renal tissue in diabetic nephropathy rats and prevents nephropathy, therefore providing a potential means for targeting therapeutic agents to kidney. 

## 5. Conclusions

MSCs transplantation reverts hyperglycemia, UTMD technique noninvasively increases the homing of MSCs to kidneys and promotes renal repair in DN rats. The key mechanism may be due to that UTMD promotes the more MSCs homing to diabetic kidney by increasing the permeability of renal interstitial capillary and VCAM-1 expression, which suppresses the inflammatory reaction by enhancing IL-10 levels, repairs the damaged glomeruli and tubules by inhibiting TGF-*β*1 expression and upregulating synaptopodin expression.
